# Comprehensive pan-cancer analysis indicates key gene of p53-independent apoptosis is a novel biomarker for clinical application and chemotherapy in colorectal cancer

**DOI:** 10.3389/fimmu.2025.1571137

**Published:** 2025-03-27

**Authors:** Jianing Yan, Jingzhi Wang, Min Miao, Yongfu Shao

**Affiliations:** ^1^ Department of Gastroenterology, The First Affiliated Hospital of Ningbo University, Ningbo, China; ^2^ Department of Radiotherapy Oncology, The First People’s Hospital of Yancheng, Jiangsu, Yancheng, China

**Keywords:** SLFN11, multiomics, pan-cancer, spatial transcriptome, immune infiltration

## Abstract

**Background:**

Schlafen11 (SLFN11) is a key gene in p53-independent apoptosis through ribosome stalling; however, systematic research has been conducted on its role in the tumor immune microenvironment, clinical application, and immunotherapy response across pan-cancer.

**Method:**

Public data were downloaded and multi-omics approaches were used to investigate the relationship between the expression level of SLFN11 and spatial position, biological function, immune landscape, and clinical application values. Cell Counting Kit-8 assay and quantitative real-time PCR were used to validate the expression level of SLFN11 and drug sensitivity in colorectal cancer samples.

**Result:**

Our study revealed that SLFN11 was downregulated in most cancers and correlated with DNA repair, the P53 pathway and immune response in tumor development progress by multi-omics analysis. Dysregulated SLFN11 is accompanied by several immune cell infiltrations and immune-related regulators, which can be a promising screening and prognostic biomarker and chemotherapy predictive target for clinical application. *In vitro* experiments proved that downregulated SLFN11 is a useful diagnostic biomarker and is linked to imatinib resistance in colorectal cancer.

**Conclusion:**

The expression level of SLFN11 has a substantial promise as a valuable biomarker for diagnosis and a predictive indicator for assessing the effectiveness of chemotherapy and immunotherapy in human cancers, which deserves further additional basic experiments and clinical trials to prove.

## 1 Introduction

Worldwide, cancer remains a major global health problem, with persistently increasing numbers of new cases and deaths in recent years (1). Although surgery, immune, and gene combination therapy have had some effect, many patients still suffer from poor or even ineffective therapy due to the emergence and spread of drug resistance (2). The biological mechanisms underlying tumor initiation and development have not been completely characterized (3).

Apoptosis is a critical mechanism in the balance between cell proliferation and cell death. As higher immunosuppression and inhibition of apoptosis occur in the tumor microenvironment, induction of tumor cell death by apoptosis is an important method for drug design. Emerging study reveals chemotherapy causing DNA damage can induce p53-independent apoptosis through ribosome stalling and downregulated Schlafen11 (SLFN11) is key gene in this progress, which is a novel cell apoptosis way with a promising applicable future (4). However, few systematic comprehensive pan-cancer studies have explored the predictive value of SLFN11 for prognosis and immunotherapy response.

In this study, we downloaded and utilized various public databases to detect differential expression levels of SLFN11 in pan-cancer and normal tissues. Meanwhile, the potential function and its influence on immune cell infiltration and chemotherapeutic effects in pan-cancer are also characterized by single-cell lines and multi-omics. *In vitro* experiments, such as quantitative real-time PCR and drug sensitivity analysis, were performed to validate the bioinformatics results.

## 2 Materials and methods

### 2.1 Public database retrieval and clinical samples acquisition

The genomic expression profiles and clinical information of pan-cancer patients were downloaded from The Cancer Genome Atlas (TCGA) database (https://genome-cancer.ucsc.edu/), and normalized RNA-seq data from the Genotype-Tissue Expression (GTEx) data portal (https://www.gtexportal.org/home/index.html). The protein levels of SLFN11 in cancers were acquired from The University of ALabama at Birmingham CANcer data analysis Portal (https://ualcan.path.uab.edu/index.html) (5). Pan-cancers contained Adrenocortical carcinoma (ACC), Bladder Urothelial Carcinoma (BLCA), Breast Cancer (BC), Cervical Squamous Cell Carcinoma and Endocervical Adenocarcinoma (CESC), Cholangiocarcinoma (CHOL), Colorectal Adenocarcinoma (COAD), Diffuse Large B-cell Lymphoma (DLBC), Esophageal Carcinoma (ESCA), Glioblastoma Multiforme (GBM), Head and Neck Squamous Cell Carcinoma (HNSC), Kidney Chromophobe (KICH), Kidney Renal Clear Cell Carcinoma (LIRC), Kidney Renal Papillary Cell Carcinoma (KIRP), Low Grade Glioma (LGG), Liver Hepatocellular Carcinoma (LIHC), Lung Adenocarcinoma (LUAD), Lung Squamous Cell Carcinoma (LUSC), Ovarian Cancer (OV), Pancreatic Adenocarcinoma (PAAD), Pheochromocytoma and Paraganglioma (PCPG), Prostate Adenocarcinoma (PRAD), Rectal Adenocarcinoma (READ), Cutaneous Melanoma (SKCM), Stomach Adenocarcinoma (STAD), Testicular Germ Cell Tumor (TGCT), Thyroid Cancer (THCA), Thymoma (THYM), Uterine Corpus Endometrial Carcinoma (UCEC), Uterine Carcinosarcoma (UCS). The Human Protein Atlas (HPA, https://www.proteinatlas.org/) online database was used to obtain protein immunohistochemistry data). The relationship between SLFN11 expression and Copy number variation (CNV), microsatellite instability (MSI) were calculated using UCSCXenaTools (v1.4.7) online tool (https://shiny.hiplot.com.cn/ucsc-xena-shiny) (6). Mutation data were acquired from cBioPortal (https://www.cbioportal.org/), an online pan-cancer genomics tool. In the methylation analysis, TSS1500 (from -200 to -1500 bp upstream of TSS), TSS200 (from -200 bp upstream of TSS), 1stExone, (the first exon), 5’UTR (5’ untranslated region [UTR]), the median value is calculated to characterize the methylation level of each sample. Spearman correlation analysis investigates the relationship between methylation level and gene expression. Clinical colorectal cancer (CRC) tissues with complete clinical data and paired adjacent non-tumorous tissues (5 cm away from the edge of CRC tissue) were collected from 30 newly diagnosed adult patients with advanced CRC who underwent gastrectomy at The First Affiliated Hospital of Ningbo University, China, between 2021 and 2023. All patients voluntarily participated in the study and underwent curative resection. All procedures were performed in accordance with the principles of the Declaration of Helsinki, and our study was approved by the Ethics Committee of the First Affiliated Hospital of Ningbo University (No. KY20210762).

### 2.2 Single-cell transcription data

Single-cell analysis was performed using the Tumor Immune Single-cell Hub 2 (TISCH2) database (http://tisch.comp-genomics.org/home/), an online tool with detailed cancer transcription data (7). CRC spatial transcriptomic data were downloaded from CRC_WholeTranscriptomeAnalysis_10x, and the R package seurat was used for computing dimension reduction and clustering analyses. All codes for transcriptomic, spatial, and statistical analyses can be found in github.com/polyak-lab/LeukocyteDCISIDC. In order to visually display the distribution of genes in different cell types in each microregion, the Sparkle database (https://www.grswsci.top) was used to perform spatial transcriptome analysis, which integrated 10xVisium sequencing to construct a pan-cancer spatial transcriptome map and adopted the SpatialPlot function in the Seurat package for visual analysis. This function can display the largest proportion of cell type information in the spatial transcriptome data in the form of an intuitive image, and generate a color-coded dot plot by combining the cell distribution and spatial location. and spearman analysis were used for data analysis (8).

### 2.3 Biological function annotation via multi-omics

TCGA patients in each cancer type were preliminarily divided into high and low groups based on the expression level of SLFN11. KEGG pathway enrichment analysis, gene ontology (GO) classification and Gene Set Enrichment Analysis (GSEA) were used to dig out biological functions which was visualized by R packages “clusterProfiler [V 4.4.4]” and “ggplot2”. P value < 0.05 and False discovery rate (FDR) < 0.25 represent statistically significant differences.

### 2.4 Immune cell infiltration landscape analysis

The TIMER 2.0, database (http://timer.cistrome.org/) was used to estimate the correlation between the expression of SLFN11 and immune cell infiltration (9, 10). 6 immune subtypes were applied to divide the TCGA samples including C1 (Wound Healing), C2 (IFN-γ Dominant), C3 (Inflammatory), C4 (Lymphocyte Depleted), C5 (Immunologically Quiet), C6 (TGF-β Dominant) (11). The relationships between the expression level of SLFN11 and immune cell infiltrations were analyzed by R packages “GSVA (1.46.0)” and “estimate (1.0.13)” with the default parameters (12). The determination of DNA genomic status as Q1-Q4 typically involves a classification system based on the quantification of certain genomic features or alterations. Based on predefined criteria, the genomic status is classified into quartiles (Q1-Q4). Spearman’s correlation analysis and ANOVA calculation were used to calculate relevance flexibly.

### 2.5 Clinical application potential analysis

TCGA and GTEx samples were used to calculate the area under the curve (AUC) value to explore tumor screening potential. Univariate Cox proportional hazard regression was employed to assess the prognostic effect of SLFN11 using SPSS software (V26.0, USA), which was visualized by forest plot using the R package ggplot2[3.3.6]. Tumor Immunotherapy Gene Expression Resource (TIGER, http://tiger.canceromics.org/) is a web-accessible portal for integrative analysis of the gene expression data related to tumor immunology, which was used to predict the relationship between the expression of SLFN11 and immunotherapy response. The Cancer Therapeutics Response Portal database (https://portals.broadinstitute.org/ctrp.v2.1/), Genomics of Drug Sensitivity in Cancer (GDSC, https://www.cancerrxgene.org/), and PRISM Repurposing dataset (https://depmap.org/portal/prism/) were employed to estimate drug sensitivity (13–15). Lower AUC values suggest higher sensitivity to treatment.

Differentially expressed genes between higher and lower SLFN11 samples in each cancer type were acquired from previous GSEA analysis, as mentioned before. The 500 most highly overexpressed or downregulated genes were identified as SLFN11-related signatures. Subsequently, 1288 compound-related signatures, which were downloaded from the database website (https://www.pmgenomics.ca/bhklab/sites/default/files/downloads), were used to calculate the matching score (16, 17). This result was summarized and visualized using the pHeatmap package (v1.0.12) in R.

### 2.6 Expression validation by quantitative real-time PCR

The separated colorectal tissues were preserved by immediate immersion in RNA save solution (Biological Industries, Israel) in an Eppendorf tube and immediately frozen by immersion in liquid nitrogen for RNA isolation. All RNAs was extracted from cells and tissues using TRIzol reagent (Ambion, Carlsbad, CA, USA) according to the manufacturer’s instructions. Two micrograms of RNA were used as a template and reverse transcribed to complementary DNA (cDNA) using a GoScript Reverse Transcription (RT) System (Promega, Madison, WI, USA) according to the manufacturer’s instructions. Subsequently, qRT-PCR was performed using GoTaq qPCR Master Mix (Promega) under the following conditions: 95°C for 5 min, followed by 40 cycles of 94°C for 15 s, 52°C for 30 s, and 72°C for 30 s. GAPDH mRNA was chosen for normalization and the primer sequences were as follows: SLFN11, forward: 5’-CCTGGTTGTGGAACCATCTT-3,’ and reverse: 5’-CTCTCCTTCTCTTGGTCTCTCT-3’; GAPDH: forward, 5’-ACCCACTCCTCCACCTTTGAC-3,’ reverse, 5’-TGTTGCTGTAGCCAAATTCGTT-3’ (18). The ΔCt or 2-ΔΔCt method was used for quantification (ΔCt= CtSLFN11- CtGAPDH, ΔΔCt=ΔCttumor-ΔCtnormal). A higher ΔCt value indicates a lower expression level (19).

### 2.7 Cell culture and Cell Counting Kit-8 assay

The human colon epithelial cell line (NCM460) and three CRC cell lines (HCT116, HT29, and SW620) were purchased from the Chinese Academy of Sciences Cell Bank (Shanghai, China). All cells were maintained by supplementation with 10% fetal bovine serum (FBS) and grown in humidified air containing 5% CO2 at 37°C in RPMI 1640 or McCoy’s 5A medium (Invitrogen, USA). Imatinib was purchased from Chemie Tek (Indianapolis, IN, USA) and prepared as a 20mM stock solution in dimethyl sulfoxide. Imatinib was serially diluted to nine different concentrations and then co-cultured with CRC cells for 12 h (20).

Cell proliferation was assessed using the Cell Counting Kit-8 kit (Beyotime Biotechnology) according to the manufacturer’s instructions. The cells were plated in 96-well plates in triplicate at approximately 4×103 cells per well and cultured in the medium. The cells were then treated with CCK-8 reagent, and the absorbance (450 nm) was measured at the indicated time points.

### 2.8 Nomogram model for CRC

In order to better predicting distal survival outcome, we included common clinicopathological factors to construct a nomogram prognostic model. R package survival [V3.3.1], and rms [V 6.3-0] were utilized to establish the 1-, 3- and 5-year overall survival time prediction nomogram model and calibration curves. Concordance index (C-index) was calculated to access the discrimination of nomogram (21). The calibration curves lie on the diagonal 45-degree line suggesting an ideal nomogram model.

### 2.9 Statistical analysis

The analyses in this study were performed using R software (version 4.2.1), SPSS software (V26.0, USA), or GraphPad (version 9.5.0). The support packages were used as previously described. P < 0.05 was considered statistically significant difference.

## 3 Results

### 3.1 Abnormal expression level of SLFN11 in pan-cancer

**Figure 1:**
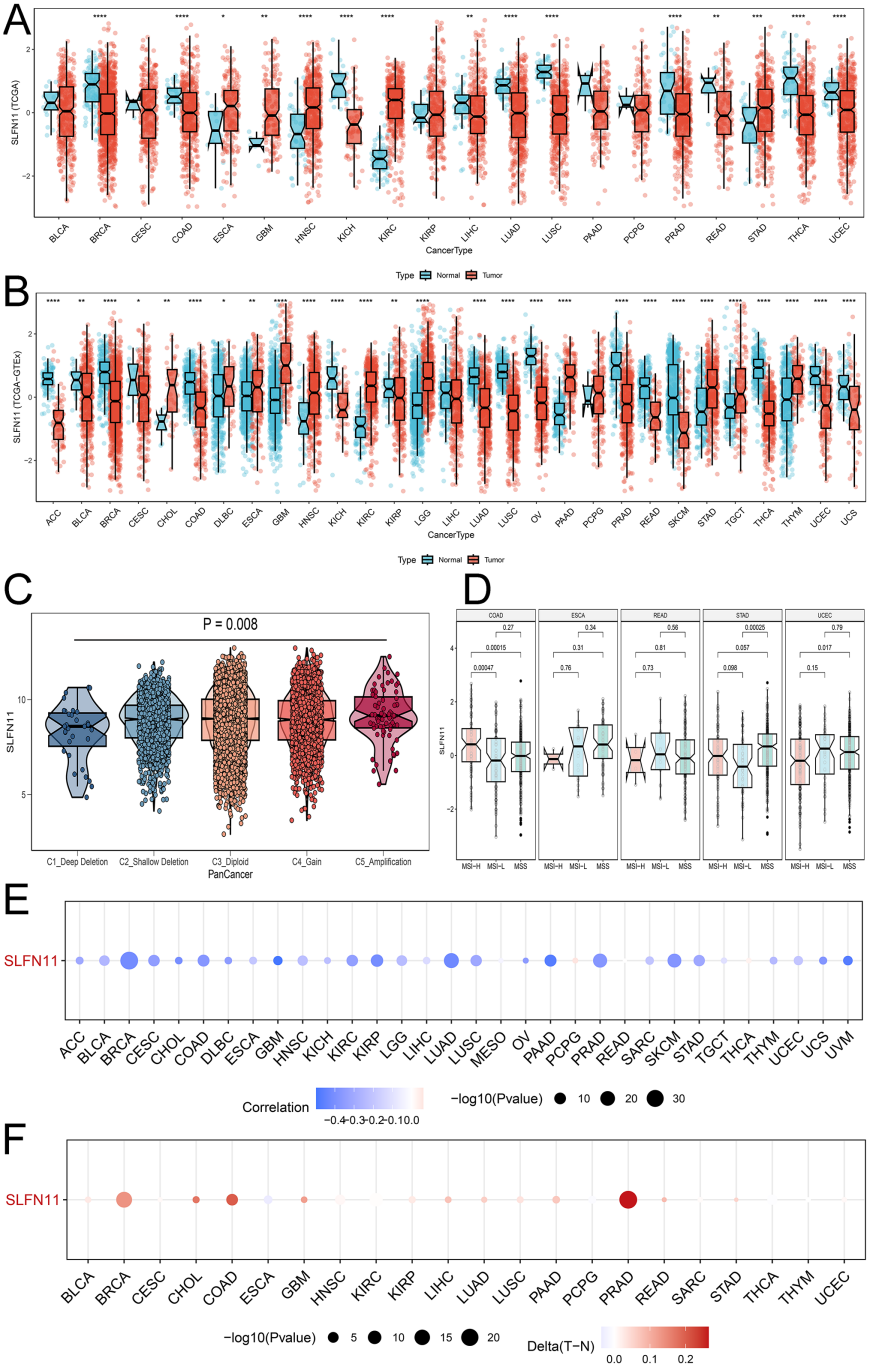
The dysregulation expression pattern of SLFN11 in pan-cancers. **(A)** The expression level of SLFN11 in TCGA cohorts. **(B)** The expression level of SLFN11 in TCGA and GTEx cohorts. **(C)** The expression level of SLFN11 in CNV groups. **(D)** The expression level of SLFN11 in MSI subtypes. **(E)** The relationship between SLFN11 expression and methylation in pan-cancers. **(F)** The relationship between SLFN11 expression and the difference of methylation in tumor and normal tissue (*P<0.05, ** P<0.01, *** P<0.001, **** P<0.0001).

To systematically detect the expression patterns in pan-cancer, we downloaded and compared the RNA expression levels of SLFN11 in TCGA database, as shown in **Figure 1A**. Our results implied that SLFN11 was significantly downregulated in the majority of cancers, such as BRCA, COAD, KICH, LIHC, LUAD, LUSC, PRAD, READ, THCA, and UCEC, which is in accordance with the dilemma of chemotherapy resistance in clinical settings. We then employed the GTEx database to increase the normal sample volume to enhance contrast, which clearly showed that SLFN11 was lower in more types of cancer (**Figure 1B**).

Copy number variation is a common variation in DNA sequences that can influence the expression and function of nearby and distal genes, causing phenotypic differences. We computed the expression of SLFN11 DNA in the five CNV types shown in **Figure 1C**. The expression of SLFN11 was the lowest in the C1_Deep Deletion group and the highest in the C5_Amplification group. Alteration frequency and phosphorylated mutation sites are shown in **Supplementary Figure 1** (**Supplementary Figure S1**). Microsatellite instability (MSI) is a symbol of mismatch repair (MMR) deficiency and is related to distal tumor prognosis. We found abnormal expression levels of SLFN11 in several MSI subtypes in COAD, STAD, and UCEC (**Figure 1D**). Furthermore, we examined the relationship between SLFN11 expression and methylation (**Figure 1E**) and the differences in methylation between tumor and normal tissues (**Figure 1F**).

**Figure 2:**
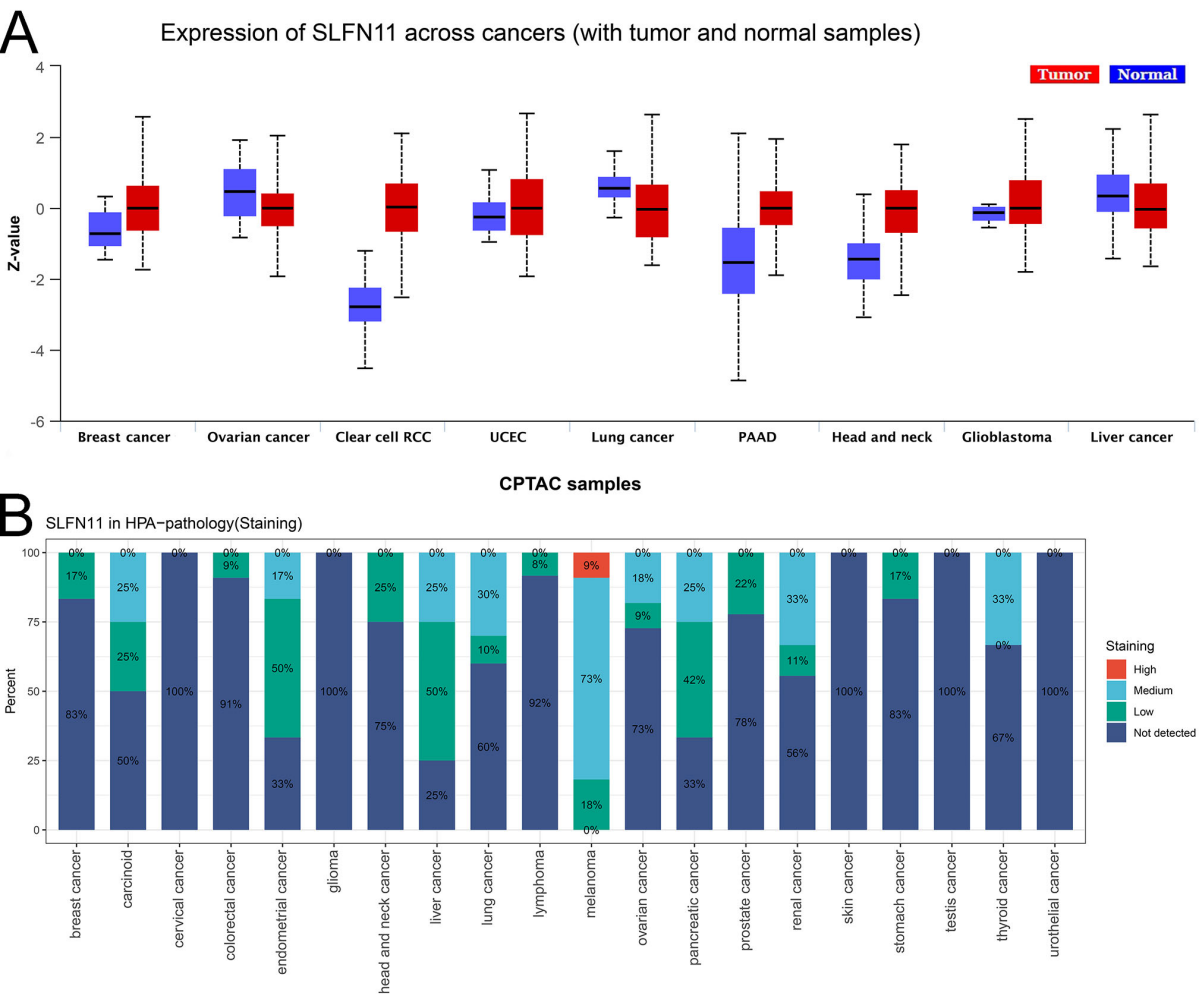
The expression of SLFN11 protein in pan-cancers. **(A)** The expression level of SLFN11 protein in CPTAC cohorts. (Red: tumor, Blue: normal). **(B)** The expression level of SLFN11 in HPA database.

To investigate the expression level of SLFN11 protein, we used the UALCAN tool and compared the differential expression of SLFN11 protein in nine types of cancers (**Figure 2A**), which showed that SLFN11 was remarkably decreased in BRAC, OV, LUAD, and LIHC. Different proportions of SLFN11 staining levels in the HPA database were calculated and are shown in **Figure 2B**. In summary, we detected and compared the expression of SLFN11 in multi-omics, and we hypothesize that SLFN11 play a key role in tumor development.

### 3.2 Single cell mapping of SLFN11 expression

**Figure 3:**
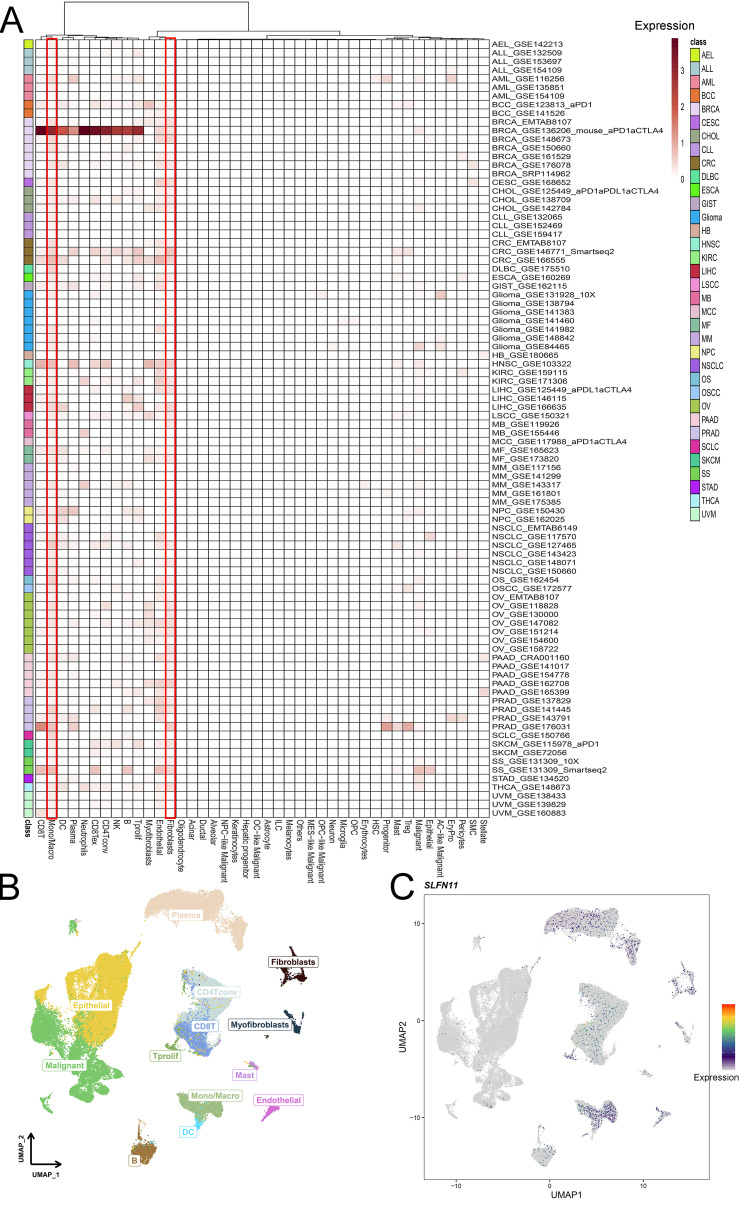
Single cell mapping of SLFN11. **(A)** The expression level of SLFN11 in single cell line in TISCH cohorts. **(B, C)** The COAD tumor microenvironment and the expression of SLFN11 in GSE166555.

**Figure 4:**
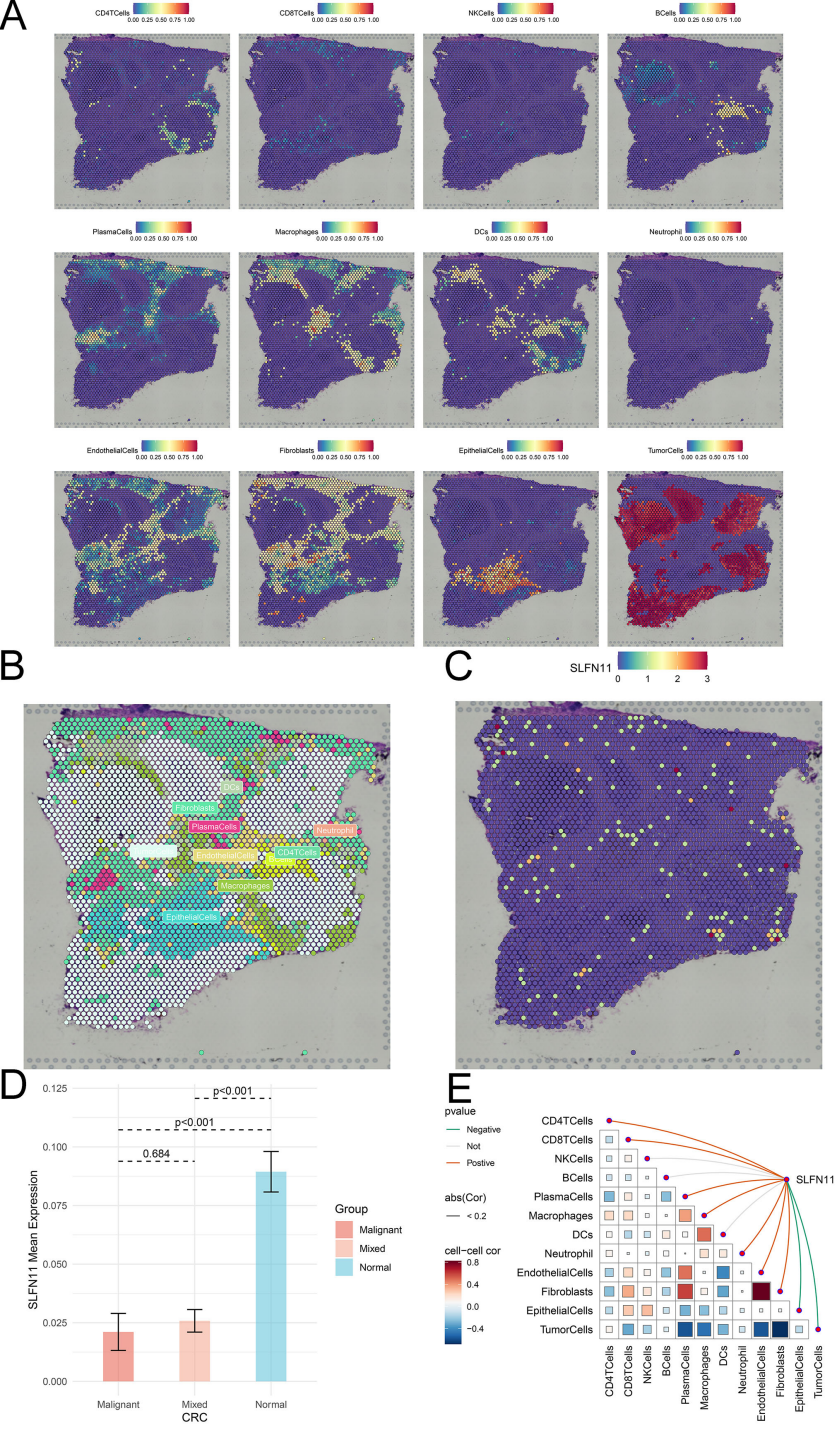
The spatial distribution of SLFN11 in CRC. **(A)** Localization of all cells after deconvolution of the spatial transcriptome in CRC sample. **(B)** Maximum value of each spot cell component after deconvolution of the spatial transcriptome. **(C)** The expression level of SLFN11 in each spot in CRC sample. **(D)** The expression level of SLFN11 in different zone of CRC tissue. **(E)** The relationship between SLFN11 expression and single cell via spatial transcriptome.

Single-cell RNA sequencing is an important method that allows mapping of gene expression to observe heterogeneity in the tumor microenvironment. We used various datasets from TISCH to comprehensively analyze the expression level of SLFN11 in a single-cell line (**Figure 3A**). SLFN11 is extensively downregulated in fibroblasts and monocytes. For example, the expression pattern of SLFN11 was clearly visualized in COAD by UMAP plots using the GSE166555 dataset (**Figures 3B, C**). Finally, the spatial transcriptomic landscape of COAD tissues was characterized using spatial transcriptomic technology (**Figures 4A–C**). It is apparent to find that SLFN11 was prominently downregulated in the malignant, mixed area (**Figure 4D**) and the relationship between SLFN11 expression and single cell was displayed in **Figure 4E** according to the spearman analysis. All of these results showed that the expression of SLFN11 focused on normal zone than mixed and tumor zone in a single sample, which implied that decreasing SLFN11 and the tumor microenvironment are inextricably linked with a high probability.

### 3.3 Biological function exploration of SLFN11 in pan-cancer

**Figure 5:**
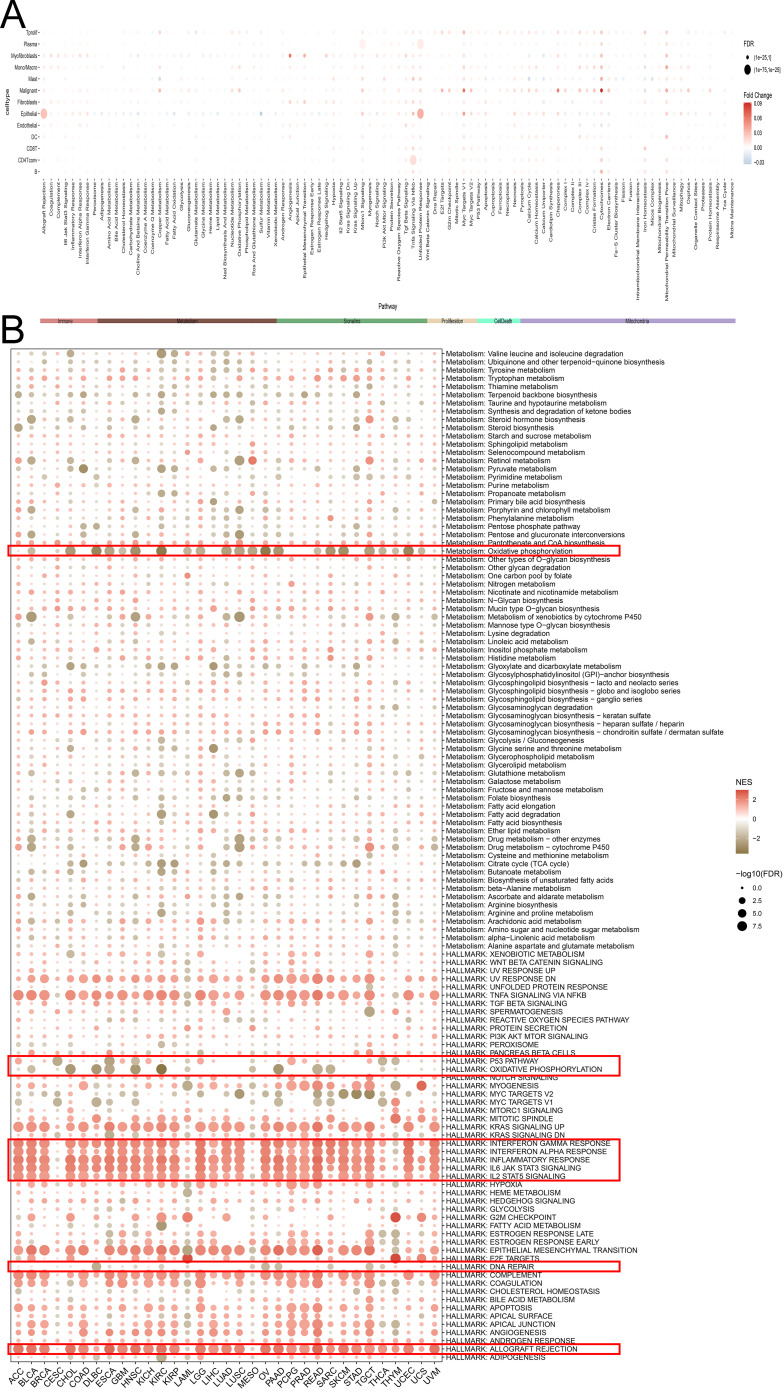
The biological function of SLFN11 in pan-cancers. **(A)** The relationship between different signal pathway and the expression level of SLFN11 in single cell. **(B)** Biological function of SLFN11 was annotated by GSEA analysis.

Given the abnormal expression levels in cancers, we further focused on exploring the biological functions of SLFN11 in cancers. We first compared and evaluated the biological functions of higher and lower SLFN11 levels based on the median value in a single cell line (**Figure 5A**). Interestingly, overexpression of SLFN11 was related to the interferon response, inflammatory response, allograft rejection, IL6/JAK/STAT3, and IL2/STAT5 signaling pathways using GESA analysis (**Figure 5B**). At the same time, downregulated SLFN11 was correlated with DNA repair, the p53 pathway, and oxidative phosphorylation. The subsequent KEGG analysis also supported this result (**Supplementary Figure S2**). All evidence suggests that SLFN11 participates in the immune response and immune infiltration during oncogenesis.

### 3.4 Immune infiltration landscape of SLFN11 in pan-cancer

**Figure 6:**
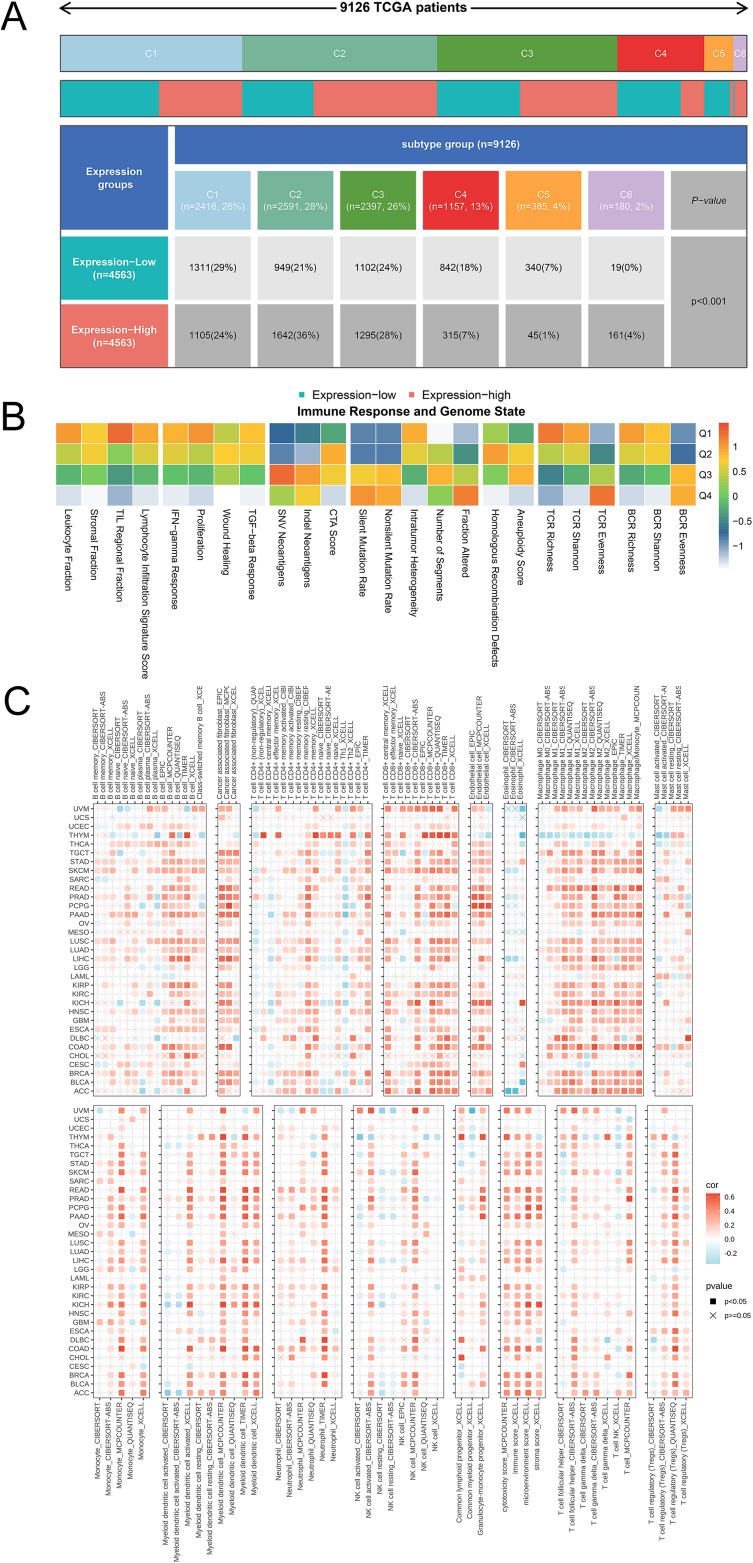
The immune landscape of SLFN11 in tumor immune environment. **(A)** The expression level of SLFN11 mRNA in 6 immune subtypes. **(B)** Diverse immune response in different DNA genomic status. **(C)** The correlation between the expression level of SLFN11 and the immune infiltration level of each immune cell using TIMER 2.0.

Considering the relationship between SLFN11 expression and an altered immune profile, we systemically investigated the immune cell infiltration microenvironment in pan-cancer. We first used TCGA database to evaluate the expression level of SLFN11 mRNA in six immune subtypes (**Figure 6A**), which indicated that lower SLFN11 always focused on the C4 group (lymphocyte depleted) and higher SLFN11 concentration in the C2 group (IFN-γdominant). Likewise, different DNA genomic status analyses revealed diverse immune responses (**Figure 6B**) in which the genomic status is classified into quartiles (Q1-Q4). Then, the TIMER 2.0 database was utilized to reveal which immune cell types could be influenced by SLFN11 expression in pan-cancer (**Figure 6C**). Our results suggest high immune cell infiltration levels of COAD and low levels of THYM, which revealed entirely different immune microenvironments in the two cancers. Meanwhile, the association between the expression of SLFN11 and immune-related regulators, such as chemokines, chemokine receptors, immunoinhibitors, and immunostimulators, was detected (**Supplementary Figure S3**), which provided bright prospects of biomarkers and immunotherapy for clinical use.

### 3.5 Estimation of clinical values of SLFN11

**Figure 7:**
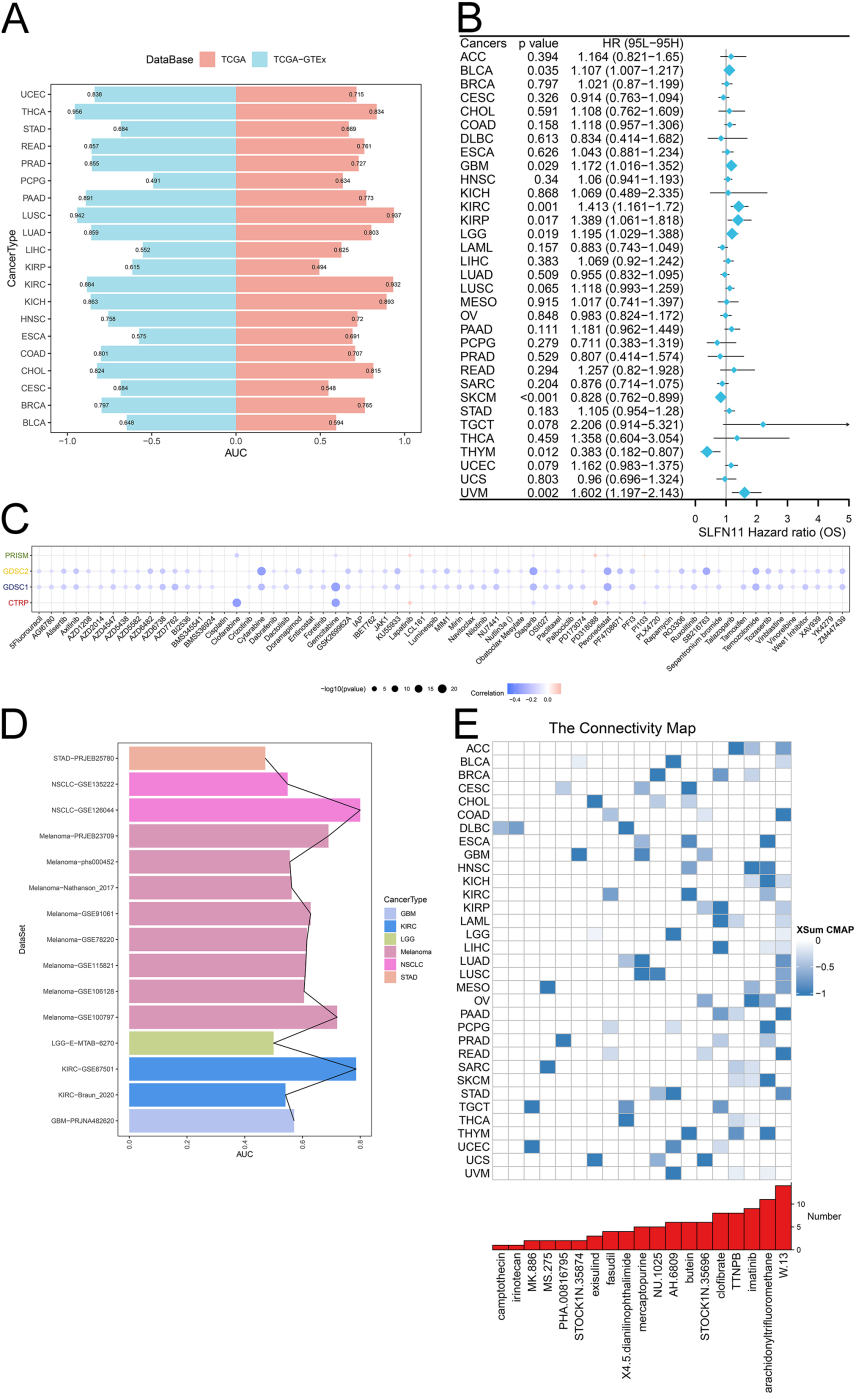
The clinical application potential of SLFN11. **(A)** The diagnostic AUC of SLFN11 in pan-cancers using TCGA and GTEx cohorts. **(B)** The relationship between the expression level of SLFN11 and overall survival time. **(C)** The correlation between the expression level of SLFN11 and several chemotherapy drugs using CTRP, GDSC1, GDSC2, PRISM databases. **(D)** The immunotherapy AUC of SLFN11 in pan-cancers. **(E)** The association between several compounds and pan-cancers.

Considering the differential expression pattern of SLFN11 and its abundant biological functions, we further evaluated the clinical application potential of SLFN11. We used TCGA and GTEx data to calculate the diagnostic AUC values, as shown in **Figure 7A**. A higher value of AUC suggested more convinced results, which implied that SLFN11 can be a novel screening biomarker in CHOL, COAD, KICH, KIRC, LUAD, LUSC, PAAD, PRAD, READ, THCA, and UCEC (AUC>0.8). Furthermore, the overall survival time of SLFN11 was also assessed and visualized in a forest plot, which implied that SLFN11 had predictive and prognostic value in BLCA, GBM, KIRC, KIRP, LGG, SKCM, THYM, and UVM (**Figure 7B**).

Chemotherapy and immunotherapy, along with surgery and radiation, are the main cancer treatments. Understanding the effects of drug resistance on both the tumor microenvironment and tumor heterogeneity is important for combining chemotherapy with immunotherapy. We investigated the relationship between the expression of SLFN11 and chemotherapy drugs using the CTRP, GDSC1, GDSC2, and PRISM databases. We found that the expression level of SLFN11 was associated with drug sensitivity to clofarabine, cytarabine, gemcitabine, olaparib, pevonedistat, and SB216763 (**Figure 7C**). Moreover, our results indicated that SLFN11 might serve as a novel target for immunotherapy, as it correlated with a higher AUC to NSCLC, Melanoma, KIRC (**Figure 7D**). Finally, several compounds with |XSum| ≥ 0.5 were identified targeted SLFN11 using CMAP analysis such as W.13 in ACC, COAD, LUAD, PAAD, READ, STAD and imatinib in HNSC, OV (**Figure 7E**). Our results comprehensively evaluated the clinical applications of SLFN11, which deserves further validation in recent clinical trial cohorts.

### 3.6 Expression level and susceptibility test validation

**Figure 8:**
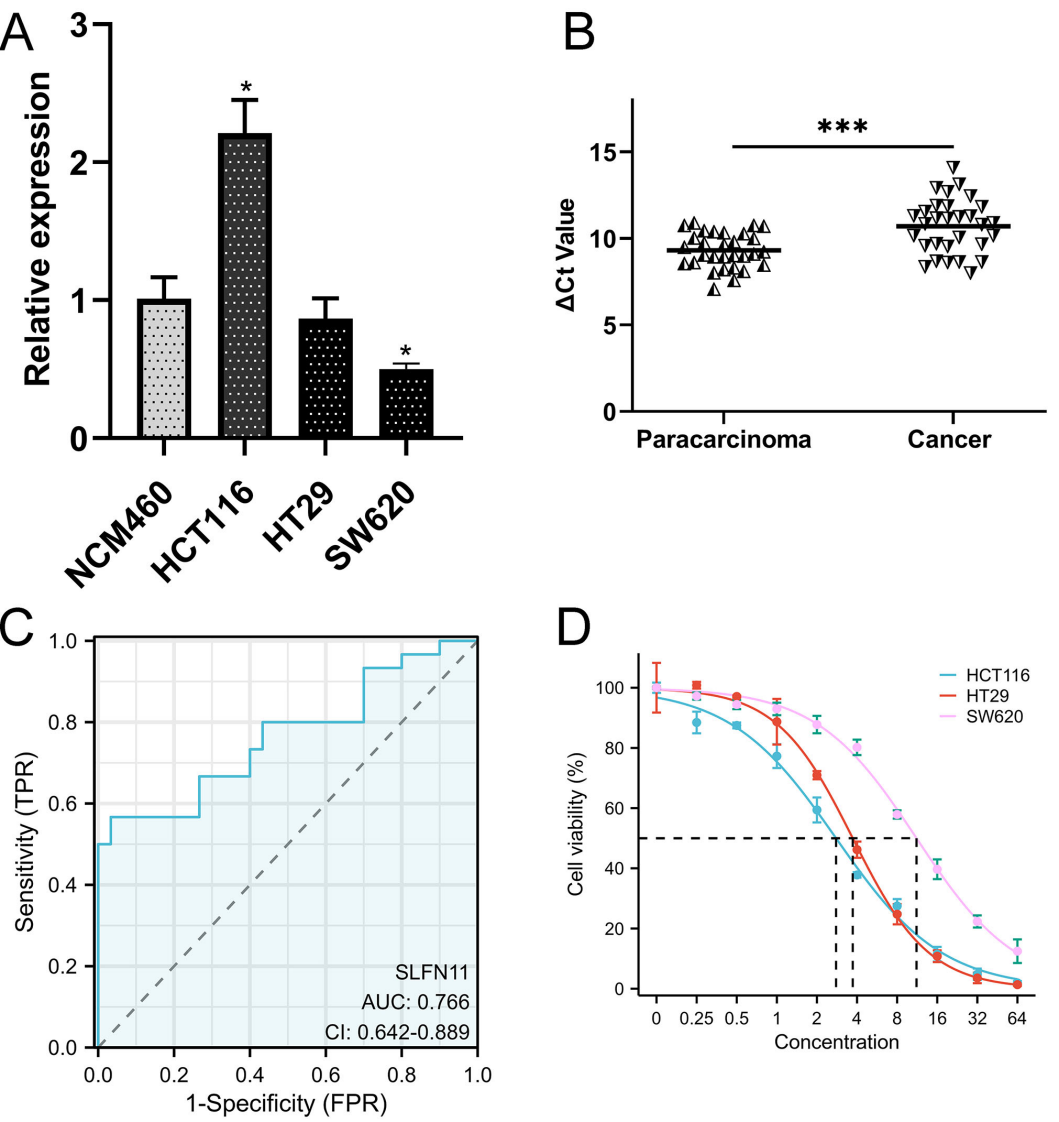
The validation of clinical application of SLFN11. **(A)** The expression level of SLFN11 in normal human colon epithelial and CRC cell line. **(B)** SLFN11 is significantly downregulated in CRC tissues. **(C)** SLFN11 has a great AUC for CRC diagnosis. **(D)** Lower expression level of SLFN11 associated with imatinib resistance by CCK-8 assay. (*P<0.05, ***P<0.001).

CRC cells and clinical CRC samples were used to validate the expression and clinical significance of SLFN11. SLFN11 was overexpressed in HCT116 cells (P <0.05) and downregulated in HT29 (P >0.05) and SW620 (P <0.05) cells (**Figure 8A**). Moreover, the expression level of SLFN11 was downregulated in CRC tissues compared to paracarcinoma tissues (**Figure 8B**, P < 0.001), which was consistent with TCGA data. The ROC curve of SLFN11 is shown in **Figure 8C**, whose diagnostic sensitivity and specificity were 66.67% and 73.33%, respectively. Furthermore, a nomogram model with several clinical characteristics and calibration curves were built for prognosis prediction in **Supplementary Figures S4A, B**. The C-index of the model was 0.764 (95% CI 0.735-0.792), which suggested this model can well guide clinical practice.

As mentioned before, imatinib could be a potential therapeutic drug based on CMAP analysis. We co-cultured CRC cells at different concentrations and detected cell viability using the CCK-8 assay. The results are shown in **Figure 8D**, and the half maximal effective concentration (EC50) was calculated. Obviously, SW620 was significantly resistant to imatinib with a higher EC50 (11.18 ± 0.48μM) compared (3.714 ± 0.102μM) to HT29 and HCT116 (2.783 ± 0.009μM), which was consistently to the expression level of SLFN11.

## 4 Discussion

The majority of tumors harbor a large number of genomic aberrations, and tumors always evolve rapidly when they get new mutations, which contributes to the development of drug resistance as intratumor genomic heterogeneity increases (22). Genome lesions trigger a series of biological responses helping cells to repair damaged DNA or induce apoptosis in which p53 tumor-suppressor protein is a vital transcription factor that modulates many DNA damage responses called the ‘Guardian of the Genome’ (23). However, the reasons why tumor cells lacking p53 can undergo apoptosis upon DNA damage remain unknown.

Recently, Boon et al. showed that DNA damage can induce p53-independent apoptosis through ribosome stalling, in which transfer RNAse SLFN11 is required for UUA stalling and global translation inhibition (4). This finding provides an important explanation for the frequent inactivation of SLFN11 in chemotherapy-unresponsive tumors. Hence, we aimed to systematically analyze the role of SLFN11 in pan-cancer to offer a new theoretical complement for future research.

In this study, the expression level of SLFN11 was determined in pan-cancers using several public databases. All genomic, RNA, and protein omics data suggest that SLFN11 is downregulated in most solid tumors. Historically, MSI has been a reliable predictive indicator of tumor prognosis and chemotherapy. MSI is present in 15% of CRC, 3% Lynch syndrome, and 12% sporadic, and is traditionally classified into three distinct groups according to Bethesda guidelines: MSI-high (MSI-H), MSI-low (MSI-L), or MSI stable (MSS) (24). Our results showed that dysregulated expression of SLFN11 was associated with MSI-H and MSI-L compared to the MSS group, which lays the foundation for further survival and drug research in COAD.

ScRNA-seq and combinations of spatial transcriptomic techniques enable the study of single-cell gene expression in complex, highly spatially organized tissues (25). As we all known, fibroblasts may regulate transmigration of monocytes and monocytes are attracted in tumor tissue by different chemokines produced by cancer cells, fibroblasts or immune cells. Fibroblasts also induce an immune response by alerting the surrounding immune cells, such as monocytes and DCs (26). Our scRNA-seq data suggest that SLFN11 is extensively downregulated in fibroblasts and monocytes, which may be associated with extensive fibrosis and poor immunotherapy response. In addition, spatial transcriptomic data provide a better understanding of transcriptome dynamics in a spatial context in CRC tissues.

Given the breakthrough in p53-independent apoptosis, the biological functions of SLFN11 require further investigation. KEGG, GSEA analyses showed that SLFN11 was tightly correlated with DNA repair, p53 pathway, and immune response. Interestingly, further analysis also revealed a close relationship between dysregulated expression levels of SLFN11 and immune cell infiltration and immune-related regulators, such as higher immune cell infiltration levels in COAD and lower levels in THYM. It has been found that upregulated SLFN11 correlates with higher immune activation in breast cancer, suggesting an important role of its immune and molecular variability in breast cancer (27). The role of SLFN11 in immune infiltration remains obscure, and we constructed a comprehensive landscape of SLFN11 in pan-cancer, investigating a critical immune context to consider when targeting immune behavior therapeutically in the future.

To date, combination chemotherapy and surgery are indispensable strategies for CRC therapy (28). Due to complicated tumor heterogeneity and the immune microenvironment, there is still a lack of predictive markers to directly use chemotherapy for CRC patients, and overuse of chemotherapy increases the economic burden on society (29, 30). Hence, the potential clinical applications of SLFN11 were evaluated. Overall, SLFN11 is a promising prognostic and screening biomarker, as well as a novel chemotherapy target. Subsequently, qRT-PCR was performed to validate the difference in expression and diagnostic value of CRC, which was in accordance with previous results. Inspired by immune response and chemotherapy analysis, we chose imatinib, an important tyrosine kinase inhibitor, to explore the relationship between the expression of SLFN11 and chemotherapeutic drug sensitivity. Recent evidence pointed out that mismatch repair, microsatellite instability and tumor mutational burden are independent biomarkers that complement each other for predicting immune checkpoint inhibitors efficacy (31). Immune checkpoint inhibitors have led to impressive, deep, and long-lasting responses in CRC in both the first-line and the refractory setting (32). Imatinib and sunitinib are the most common clinically used small molecule inhibitors against mutant PDGFRA (33). However, the relationships between imatinib sensitivity and SLFN11 level as well as CRC are still unknown. As a hot drug in clinical chemotherapy, imatinib has a promising future in clinical application so we are interested about the curative effect about imatinib in CRC. Meanwhile, the safety about W.13 is vague and it is difficult to buy and set its titer. Thus, we choose imatinib for further validation. SLFN11 was prominently downregulated in SW620 cells compared with that in NCM460 cells. Although the difference did not reach statistical significance, we still observed a trend of lower expression levels in HT29 cells. Interestingly, the EC50 showed an obvious upward trend as SLFN11 gradually decreased, which demonstrated that lower SLFN11 levels were associated with higher resistance to chemotherapy drugs and higher tumor cell viability in CRC. SLFN11 may be a novel diagnostic biomarker and chemotherapy predictive target for CRC.

Our study still exists some limitations and imperfection. For example, the difference did not reach statistical significance in HT29 cells in drug sensitivity experiment. As limited by funds and conditions, more basic experiments in molecular biology such as SLFN11 knocking-down and reserve needs to be performed in the future. Meanwhile, conducting a longitudinal study to track the long-term outcomes of the interventions or phenomena observed in our research would provide deeper insights into the durability and potential late effects of the findings.

In conclusion, our study revealed that SLFN11 is downregulated in most cancers compared to normal tissues, which correlates with DNA repair, the P53 pathway and immune response in tumor development progress by multi-omics analysis. Notably, the expression level of SLFN11 shows substantial promise as a valuable biomarker for the diagnosis and assessment of the effectiveness of chemotherapy and immunotherapy in human cancers, which deserves further basic experiments and clinical trials.

## Data Availability

The datasets presented in this study can be found in online repositories. The names of the repository/repositories and accession number(s) can be found in the article/**Supplementary Material**.
