# Supplementary material for: Comprehensive pan-cancer analysis indicates key gene of p53-independent apoptosis is a novel biomarker for clinical application and chemotherapy in colorectal cancer
Source: Front Immunol. 2025 Mar 27;16:1571137. doi: 10.3389/fimmu.2025.1571137 (PMC11983446; doi:10.3389/fimmu.2025.1571137)
Supplement: Supplementary file 1 [file DataSheet1.docx]

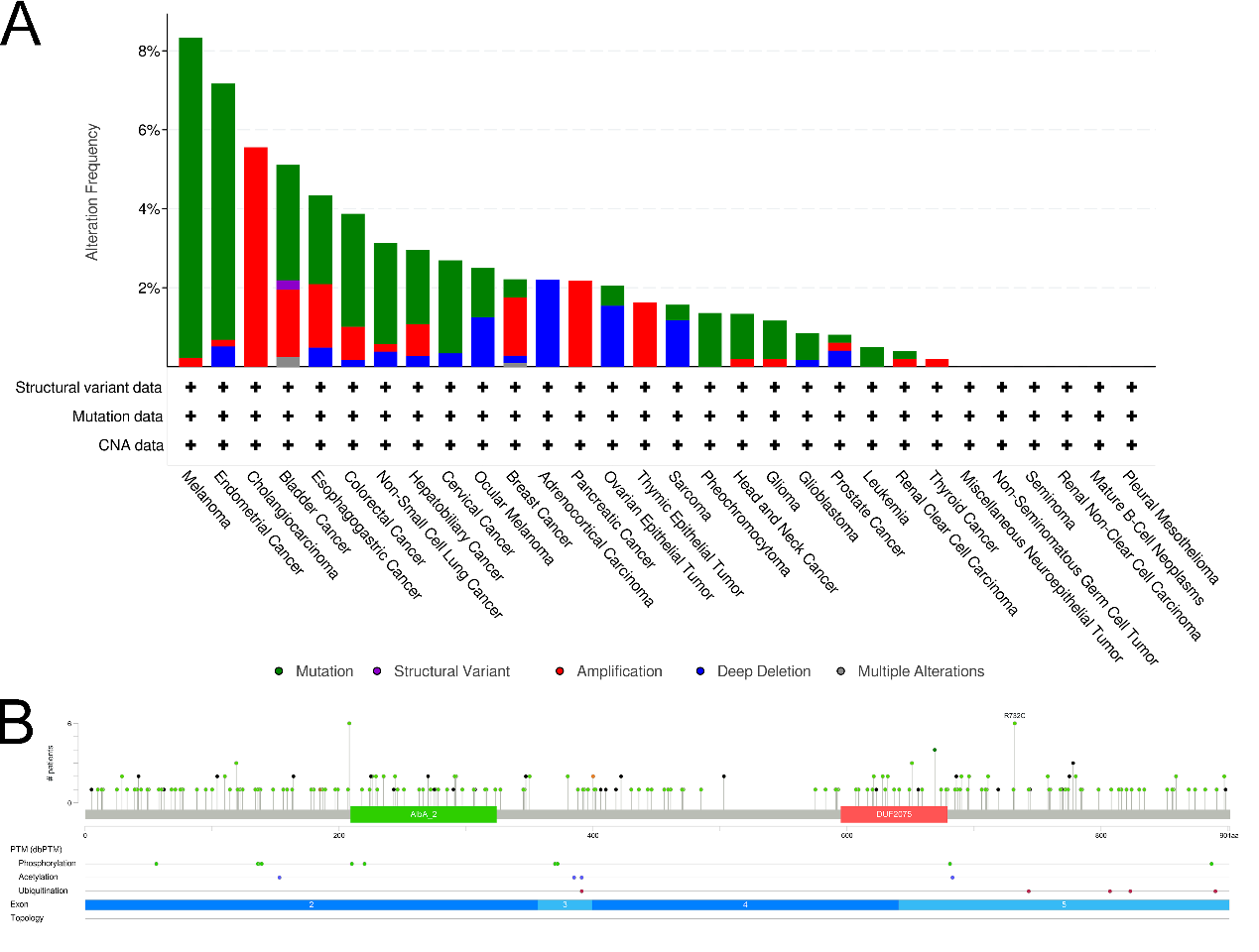
**Supplementary Figure 1 The alteration frequency and phosphorylated mutation sites of SLFN11**


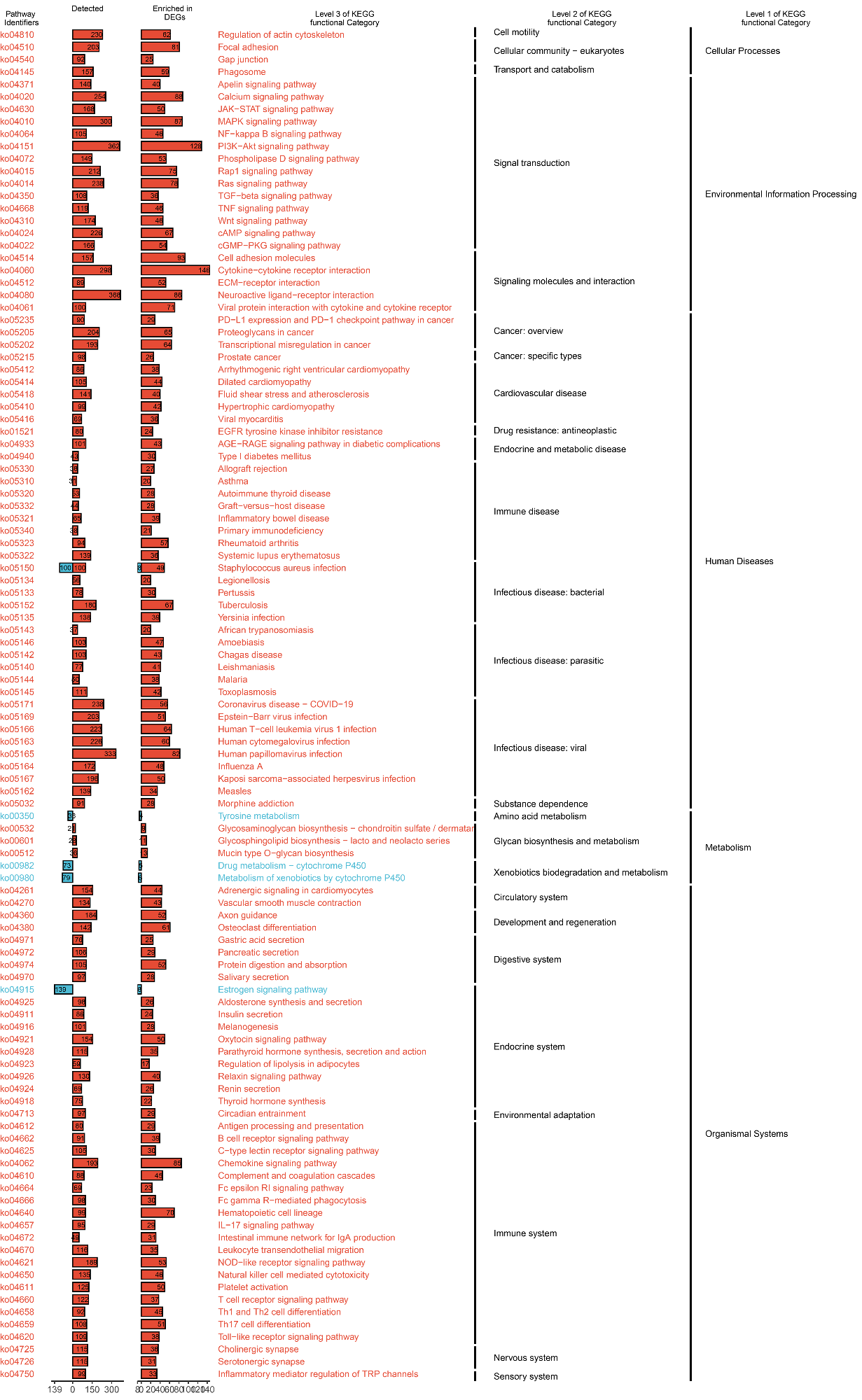
 **Supplementary Figure 2 KEGG analysis**

**
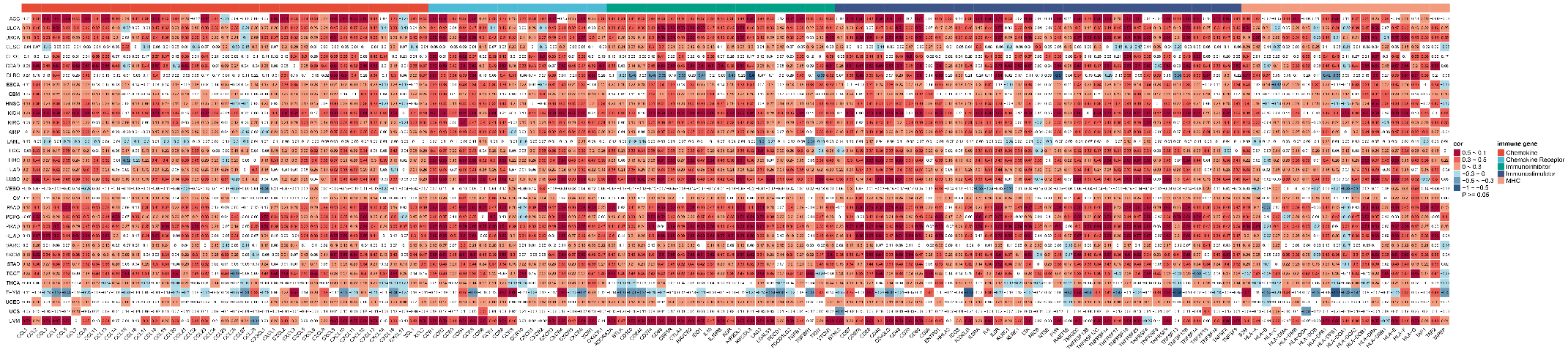
**

**Supplementary Figure 3 The expression between SLFN11 and immune-related regulators**

**Supplementary Figure
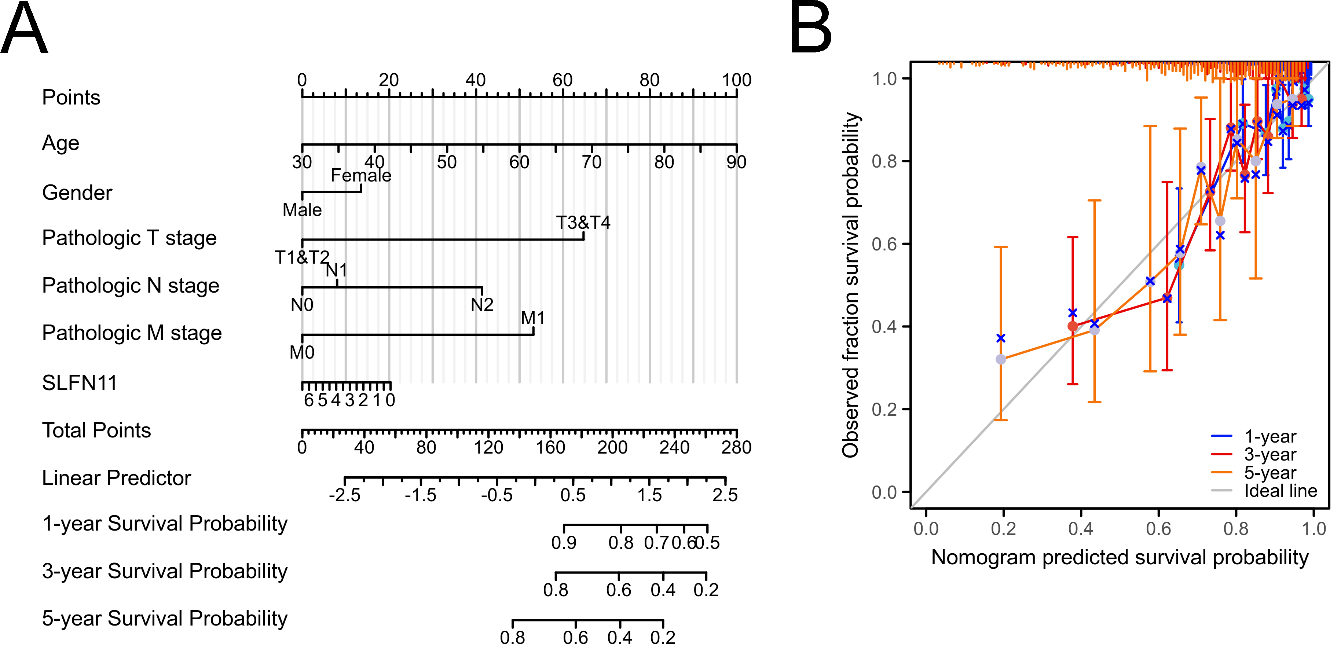
4 Nomogram model and calibration curve**
